# Mortality from Tuberculous Phthisis

**Published:** 1901-07-27

**Authors:** 


### Mortality from Tuberculous Phthisis.

In a paper read by Dr. John Tatham, F.R.C.P., tables are given which show that between the years 1851 and 1899 every age, with a few trifling exceptions, has experienced a decrease in the mortality ascribed to phthisis. In every case the mortality in 1895-99 was lower than it had been in 1851-60, but the rate of decrease has varied widely, and has been much greater among females than among males. The reduction shown by the tables may not, however, be wholly real, and may be due in part to the vaguer statement of causes of death in the earlier period; as, for instance, by the habitual use of the terms "consumption" and "decline" to describe any lingering disease of the lungs attended by "wasting." The figures, therefore, must be taken for what they are worth.

Tracing backwards through all these years the incidence of the maximum mortality from phthisis we find that it has not always been at the ages from 45 to 55 for males and from 35 to 45 for females, as it is at present. In the decade 1851-60 phthisis mortality was at its highest among males at ages from 20 to 25 and among females at ages from 25 to 35. Thus the period of maximum phthisis mortality has been postponed in both sexes. In other words, either the saving of life has been greater at the ages which were formerly most liable to phthisis than at the ages immediately following, or persons specially liable to phthisis have lived longer than they would have done under the earlier conditions.

